# Perinatal and neonatal outcomes among women with multimorbidity during pregnancy globally: a systematic review

**DOI:** 10.1186/s12887-026-06706-9

**Published:** 2026-03-18

**Authors:** Mary Hewitt, Adhithi Sreenivasan, Sunjuri Sun, Soha El-Halabi, Claudia Hanson, Veronika Tirado

**Affiliations:** 1 Department of Global Public Health, Karolinska Institutet, Stockholm, Sweden; 2 London School of Hygiene and Tropical Medicine, London, UK; 3 Swedish centre for impacts of climate extremes (climes), Karolinska Institutet, Stockholm, Sweden

**Keywords:** Multimorbidity, Pregnancy, Maternal health, Perinatal outcomes, Neonatal outcomes, Systematic review

## Abstract

**Background:**

Multimorbidity during pregnancy, the coexisting of two or more chronic health conditions, is linked to negative impacts on foetal development, adverse birth outcomes, and increased risk of future health problems for mother and child. While existing research has explored single chronic conditions in relation to pregnancy outcomes, there remains a significant gap in understanding how multimorbidity influence maternal and neonatal health. This review aims to identify, assess, and synthesise literature on multimorbidity during pregnancy and its adverse perinatal and neonatal clinical outcomes to inform future research and public health interventions.

**Methods:**

We conducted a systematic review of perinatal and neonatal outcomes, following the PRISMA 2020 guidelines. Embase, Web of Science, and Medline databases were searched to find relevant cohort and case-control studies published (January 1, 2015-March 3, 2025). There were no restrictions on setting or language. A narrative synthesis was conducted to summarise the existing findings between multimorbidity during pregnancy and adverse outcomes in offspring.

**Results:**

Of 7,531 publications screened, 54 articles were included. Most studies (*n* = 28) reported on pregnant women with multiple physical non-communicable diseases (NCDs), followed by those with infectious diseases (*n* = 11), two or more mental health conditions (*n* = 5), environmental exposures (*n* = 3), comorbid physical NCDs and mental health conditions (*n* = 5), and substance use (*n* = 2). The most common outcomes reported were preterm birth (*n* = 30), neonatal intensive care unit admission (*n* = 15), low Apgar scores (*n* = 13), neonatal mortality (*n* = 13), and small-for-gestational age (*n* = 13). This systematic review also revealed that there is variance in how multimorbidity is defined and how neonatal outcomes are measured and recorded, including the terminology used, values for adverse outcomes, and the points at which the outcomes were measured.

**Conclusions:**

This review identified adverse outcomes associated with multimorbidity during pregnancy and highlighted the need to shift research priorities towards multimorbidity research, especially in lower-income countries. A concrete definition of multimorbidity and a globally standard set of measurements to be recorded at birth are needed to facilitate research that is generalisable across contexts. By identifying pregnancy outcomes in women with multimorbidity, vulnerable populations can be targeted for more effective interventions.

**Supplementary Information:**

The online version contains supplementary material available at 10.1186/s12887-026-06706-9.

## Background

Multimorbidity is a phenomenon that is becoming increasingly common, posing a threat to patients and healthcare systems around the world [1, 2]. Multimorbidity differs from comorbidity both clinically and by definition. While comorbidity refers to an entity that exists in addition to a condition of interest, multimorbidity is the coexistence of two or more conditions, infections, or exposures without one taking precedence [3]. In practice, comorbidities refer to additional conditions relevant to specialists who intend to treat a specific disease or organ system. However, multimorbidity is considered in a holistic assessment of an individual, allowing providers to act according to the patient’s preferences for their treatment. Although these terms have been used as interchangeably, research efforts have increasingly sought to clarify their differences, distinguishing multimorbidity and comorbidity as separate concepts [4].

The prevalence of pregnant women with multimorbidity is increasing rapidly, affecting millions of women, especially in lower socioeconomic groups and older maternal populations [1, 5]. Pregnant women with multimorbidity face unique challenges due to the interaction of multiple conditions and treatments [5]. During pregnancy, women undergo physiological changes that can cause dramatic changes to cardiovascular function, nutritional metabolism, and body composition, which can spur the development of new conditions during pregnancy or heighten the effects of preexisting diseases. Pregnancy adds complexity to managing multimorbidity, as various conditions can develop or worsen during pregnancy, creating barriers to timely care [6]. The impact of multimorbid conditions extends beyond pregnancy as they can have permeating effects for both the development of future conditions in the mother and future generations [7]. Approximately 14% of women globally experience gestational diabetes during pregnancy, which has been correlated with a ten-fold increased risk of developing type-2 diabetes and cardiovascular disease later in life [7, 8]. The mechanisms linking maternal multimorbidity to adverse pregnancy outcomes are likely multifactorial, involving intertwined physiological and social pathways.

Multimorbidity during pregnancy has been shown to have adverse effects on perinatal characteristics and neonatal outcomes [9]. These outcomes include a wide range of maternal, foetal, and neonatal health indicators that are influenced by biological, psychological, and environmental factors during pregnancy, childbirth, and the postpartum period [10]. Adverse perinatal outcomes include complications, such as preterm birth, congenital anomalies, low Apgar scores, or abnormal size at birth, such as small-for-gestational age (SGA), large-for-gestational age (LGA), foetal growth restriction (FGR), and low birth weight [11]. Similarly, neonatal outcomes include infections, jaundice, hypoglycaemia, hyperbilirubinemia, respiratory distress syndrome, including asphyxia or the need for respiratory support after birth, and admission to the neonatal intensive care unit (NICU) [12]. Adverse perinatal and neonatal outcomes may be caused by maternal diseases, exposures, or deficiencies that affect foetal development or obstetric complications before or during childbirth [6].

Understanding the nature of these conditions is important to guide a comprehensive mapping of perinatal and neonatal outcomes, allowing a holistic evaluation of maternal and infant health during and after birth to be conducted. While other systematic reviews have studied the association specified adverse neonatal outcomes of interest with multimorbidity during pregnancy [9], this systematic review intends to identify all the adverse perinatal and neonatal outcomes reported in the literature within the first year of life without limiting the scope to predetermined outcomes, and to describe how multimorbidity during pregnancy has been defined. This systematic review is necessary to assess the neonatal and perinatal outcomes associated with multimorbidity during pregnancy without restricting outcomes or setting to guide future research and policies for pregnant women with multimorbidity.

## Methods

The review follows the guidelines outlined by the Preferred Reporting Items for Systematic reviews and Meta-analyses (PRISMA) 2020 statement [see Additional file 1] [13]. The objective for this review is to identify, assess, and synthesise existing literature that describes the definition of multimorbidity during pregnancy and its adverse perinatal and neonatal clinical outcomes, PROSPERO ID: CRD420251002023.

### Search strategy

A search strategy [see Additional file 2] was designed and conducted using the advanced search feature in Embase, MEDLINE, and Web of Science to complete comprehensive searches identifying peer-reviewed papers published between January 1 st, 2015, and March 3rd, 2025. The search was restricted by date to collect the most recent available data but was not restricted by language or setting to assess global trends in outcome variation and analyse differences in settings within different regions and with varying socioeconomic levels.

### Selection criteria

The articles retrieved from each database were filtered via Covidence software [14], and 5,435 duplicates were removed, resulting in 7,572 prospective articles to be screened for eligibility (Table 1). A pilot screening was conducted but these decisions were not included in the final screening. Screening was conducted by a primary reviewer (MH). Data extraction and quality assessment of the articles were performed independently by two reviewers (MH and VT or MH and SEH) throughout the process. To ensure accuracy and consistency, a second reviewer (VT) cross-checked 10% of the prospective articles for eligibility to validate the screening and data extraction process. Discrepancies were resolved through discussions with the research team. The full texts of all papers, with the exception of three that could not be retrieved, were requested in English through the Karolinska Institutet library. Any papers originally published in languages other than English were translated using the Google Translate browser extension (version: 2.0.16) or DeepL Pro (Advanced version). Articles lacking reported outcomes of interest were excluded during title and abstract screening, as they failed to meet the eligibility criteria. Full-text articles unobtainable after reasonable efforts were also excluded from inclusion.

**Table 1:** Eligibility criteria

	Inclusion Criteria	Exclusion Criteria
Population	Pregnant women - studies with infants as the primary population of interest were included if multimorbidity during pregnancy was analysed in relation to outcomes within the first year of life.	Non-pregnant women, men, or children.
Exposure	Multimorbidity or multiple conditions, including infectious diseases, NCDs*, mental health conditions, environmental exposures, and substance use disorders.	Single chronic conditions, without considering the impact of multimorbidity.
Outcome	Neonatal and perinatal outcomes (e.g. preterm birth, low birth weight, neonatal mortality) within one year after birth.	No relevant perinatal or neonatal outcomes reported within the first year of life.
Design	Studies of observational or interventional design with more than 100 participants, published after January 1 st, 2015, in any language or location.	Publications with unclear methodologies or incomplete data, or of non-observational study designs such as reviews, editorials, and letters to the editor. Animal studies and laboratory-based research were excluded to maintain the scope of this study.

*NCDs defined in our review as: physical conditions not caused by infectious agents that often cause chronic health consequences and require long-term treatment or care. Physical NCDs were separated from mental health conditions to provide additional analysis on how these exposures impact neonatal and perinatal outcomes

### Data extraction

Extraction was completed independently (MH, VT, SEH), including the title, author, year of publication and journal in which it was published, research question and specific aims, population of interest, hypothesis, methods, and results. A narrative synthesis approach was used to summarise the results presented in the literature, including conditions constituting multimorbidity, reported neonatal outcomes, and contextual factors relevant to the study population [15]. To facilitate synthesis of the results, the conditions constituting multimorbidity were categorised into NCDs, infectious diseases, psychological morbidities or mental health conditions, environmental exposures, and substance use disorders.

### Narrative synthesis

The narrative synthesis approach in this review is intended to summarise the study characteristics and reported outcomes based on each category of multimorbidity, explore patterns within categories, and assess study quality and contextual impact according to the Economic and Social Research Council (ESRC) framework [16]. The studies were organised in their categories of multimorbidity, and the significance reported in studies were tabulated, and the results were used to summarise patterns within categories of multimorbidity. A meta-analysis was not conducted due to variation in exposures, outcomes, and definitions of multimorbidity. The association between multimorbidity and the adverse perinatal and neonatal outcomes of interest were extracted from the studies. A forest plot was not generated due to substantial heterogeneity in effect measures (i.e., adjusted odds ratio, adjusted risk ratios, p-values without confidence intervals, and unadjusted proportions).

### Study quality and risk of bias

A quality assessment and critical appraisal of the included studies have been completed (MH, VT, SEH) using validated tools appropriate for each study design and to evaluate selection bias and comparability of the study groups and outcome measurements. The Newcastle-Ottawa Scale (NOS) [17] [see Additional file 3] for assessing the quality of non-randomised studies has been used to judge the studies on the selection of the study groups, comparability of the groups, the outcome of interest, participant follow up, and the study’s comparability within the target population. The NOS scores studies up to 9* across three domains: Selection (up to 4*), Comparability (up to 2*), and Outcome (up to 3*). Although there is variance in the NOS results for the included studies, all studies scored a 6* or higher and the majority of the studies scored a 9*, which indicates the highest quality.

For the purpose of this review, these scores have been split into two categories: those that scored 6–7 were labelled moderate studies while those that scored 8–9 were labelled high quality studies. Of the cohort studies, 38 scored a nine, indicating the highest quality [18–55]. Five of these studies scored an 8, which also indicated high quality with only one aspect of the checklist missing [56–60]. Four studies scored a 7, which showed moderately high study design, transparency, and analysis [61–64], while one study showed a 6, which was also a moderate score [65]. The case-control studies were graded on the same scale, with two studies scoring a 9 [66, 67]. One study was given a score of 8 [68], while two studies received a 7 [69, 70]. Finally, one case-control study was given a score of 6 [71]. Discrepancies in scoring were resolved through discussions among the reviewers, with a fourth reviewer involved when necessary to reach consensus.

### Ethics

Transparency was prioritised throughout the review process, and efforts were made to minimise bias by employing a systematic screening process. As this review was intended to influence future research directions and policy making, any conflicts or biases have been disclosed, and reflexivity of the authors has been considered.

## Results

**Fig. 1:**
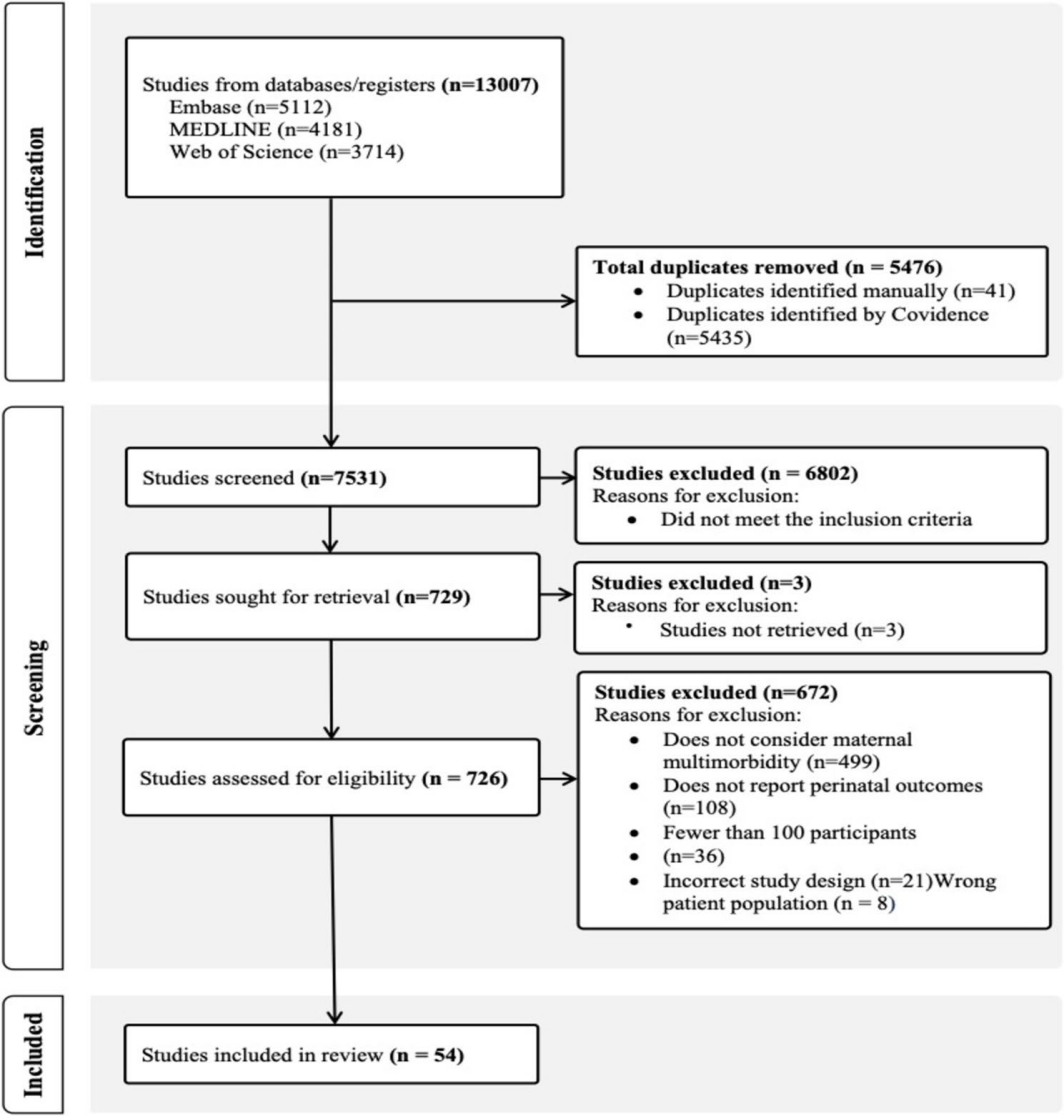
PRISMA flow diagram for study inclusion

The search resulted in 5,112 articles in Embase, 4,181 articles in MEDLINE, and 3,714 articles in Web of Science (Fig. 1). After compiling the search results from the three databases and removing duplicate records, 7,531 individual studies were screened for relevance based on the inclusion criteria. Screening titles and abstracts resulted in the identification of 729 articles to be retrieved for full text review, and upon applying the eligibility criteria, 54 articles were selected for inclusion in the review.

### Study characteristics

**Fig. 2:**
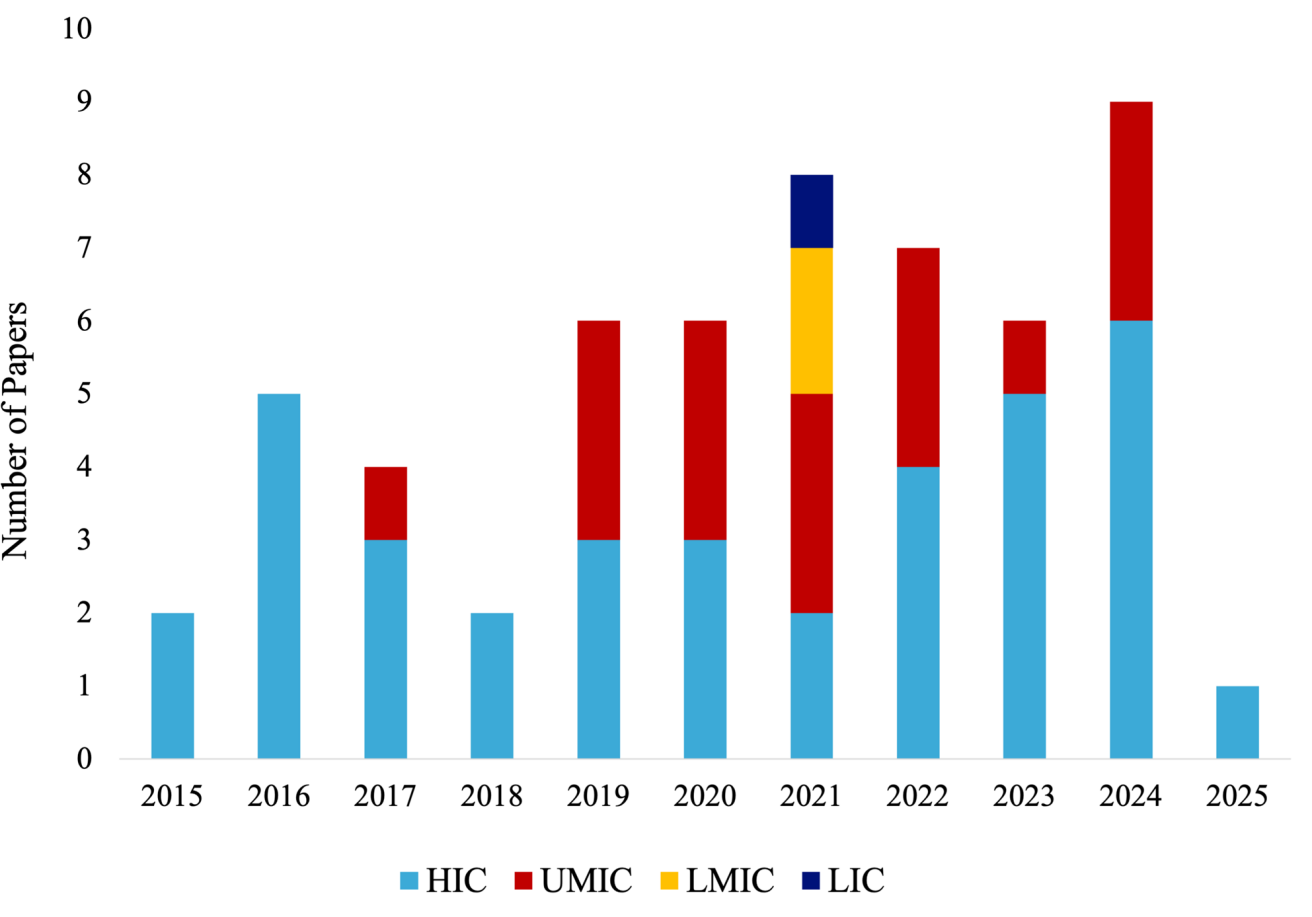
Number of included papers by publication year and World Bank income classification

The studies in this review represented 20 countries across eight world regions, as defined by the United Nations (UN) Sustainable Development Goals (SDG) regions [72] − for the purpose of this article, Northern America and Europe have been divided into two separate regions. Most studies were conducted in Northern America, with 21 papers included from this region [18, 20, 25, 26, 31, 36, 37, 41, 43, 47, 49, 51, 53, 56, 57, 59–61, 63, 67]. This was followed by Europe (*n* = 9) [19, 21, 28, 29, 33, 64–66, 69], Eastern and South-Eastern Asia (*n* = 8) [34, 35, 38–40, 54, 55, 71], and Sub-Saharan Africa (*n* = 4) [30, 45, 58, 68]. The regions of Australia and New Zealand, Northern Africa and Western Asia, and Latin America and the Caribbean each had three included papers [24, 27, 42, 46, 48, 50, 52, 62, 70]. Central and Southern Asia had the fewest number of studies included, with two included reports [23, 44]. The distribution of publication settings and the year of publication is shown in Fig. 2.

This figure shows the number of papers included in the study organised by their year of publication (2015–2025) and the World Bank income classification of the countries studied [73]. Colours indicate income classification as follows: light blue represents high-income countries (HIC), red represents upper-middle-income countries (UMIC), yellow represents lower-middle-income countries (LMIC), and dark blue represents low-income countries (LIC).

While most papers were conducted in a singular country, one study included participants from both South Africa and the United States [57, 72]. Another included data from 43 low-income countries and lower middle-income countries (LMICs) in six UN world regions [22]. This was the only report included that utilised data from low-income countries. Most studies included in this review (Table 2) were conducted in high-income countries (HIC) (*n* = 36) [18–21, 24–29, 31–34, 36, 37, 41–43, 47, 49–51, 53, 56, 57, 59, 61–67, 69]. Eighteen studies utilised data from middle-income countries, with seventeen studies included from upper middle-income countries [23, 30, 35, 38–40, 45, 46, 48, 52, 54, 55, 57, 58, 68, 70, 71] and only one study from a lower middle-income country [44].

**Table 2:** Characteristics and summary of studies of multimorbidity among pregnant women

Reference	Country	Study Period	Data source	Number of participants (women or births)	Number of conditions defining multi-morbidity	Type/included conditions	Number of adverse outcomes reported	Type/included adverse outcomes
Allen, 2016 [18]	USA	1996–2010	Registry	21,857	2	- Preeclampsia - liver dysfunction	6	- Preterm birth, - infant death, miscarriage, - stillbirth, - overall foetal mortality rate, - NICU admission
Aubry, 2019 [19]	Switzerland	2005–2016	Registry	2,170	3	- Obesity - diabetes, - preeclampsia, or hypertension	8	- Apgar score ≤ 7 at 5 min, - early neonatal death, - fracture of the clavicle, - NICU admission, - macrosomia, - neonatal hypoglycaemia, - preterm birth, - respiratory distress of newborn
Azcoaga- Lorenzo, 2023 [21]	Scotland	2014–2018	Registry	30,557	≥ 2	- Preexisting long-term physical or mental health conditions	1	- Preterm birth
Bapayeva, 2022 [23]	Kazakhstan	2012–2017	Medical records	323	2	- Diabetes mellitus - obesity	1	- Preterm birth
Bartáková, 2017 [69]	Czechia	2011–2013	Registry	432	2	- Gestational diabetes mellitus - hypertension	3	- Apgar score, - abnormal base excess (BE) or umbilical cord blood pH, - foetal death
Belsti, 2024 [24]	Australia	2016–2021	Registry	48,502	2	- Pre-existing and/or - pregnancy-related medical conditions	4	- Preterm birth, - foetal death, - congenital defects, - newborn admission to a neonatal special care nursery and/or NICU
Brakewood 2024 [25]	USA	2002–2013	Medical records	5,416,171	≥ 2	- Chronic hypertension, - renal disease, - autoimmune disease, - asthma, - cardiac disease, - bleeding disorder, and/or clotting disorder	7	- Neonatal morbidity, - foetal growth restriction, - macrosomia, - shoulder dystocia, - gestational age at delivery (average), - NICU admission, - neonatal death
Bröms, 2016 [66]	Sweden	2006–2010	Registry	246	≥ 2	- Inflammatory bowel disease, - hypertension, - diabetes or systemic disease	1	- Preterm birth
Chaalan, 2024 [27]	Qatar	2021–2021	Medical records	1,892	≥ 2	- Obstetric Comorbidity Index [74]	6	- Foetal morbidity, - Apgar score, - low umbilical artery pH (< 7,2), - hypoxic ischemic - encephalopathy, - NICU admission
Chen, 2022 [28]	Finland	1996–2014	Registry	1,097,753	2	- PCOS - diabetes mellitus	3	- Preterm birth, - LGA, - SGA
Chen, 2023 [29]	Finland	2004–2014	Registry	652,732	≥ 2	- Hypertensive disorders (gestational hypertension, - chronic hypertension, or - pre-eclampsia) and PCOS	4	- Preterm birth (< 37 weeks), - very preterm birth (28–31 weeks), - SGA, - LGA
Conti- Ramsden, 2019 [30]	South Africa	2015–2016	Registry	1,547	2	- Preeclampsia - acute kidney injury	2	- Stillbirth, - neonatal death
Cozzi, 2024 [56]	USA	2016–2018	Medical records	337	≥ 2	- Chronic hypertension, - pre-gestational diabetes mellitus, - systemic lupus erythematosus, - chronic kidney disease	11	- Birth weight, - NICU admission, - mechanical ventilation, - cardiopulmonary resuscitation, - necrotizing enterocolitis, - intraventricular haemorrhage, - respiratory distress syndrome, - arterial cord pH < 7.1, - Apgar score at 5 min, - hypoxic-ischemic encephalopathy, - neonatal death
Dudley, 2017 [32]	USA	2011–2014	Medical records	1,257	2	- Obesity or morbid obesity - diabetes mellitus	7	- Composite neonatal morbidity, - RDS, - NICU admission, - stillbirth, - neonatal death, - macrosomia, birthweight
Eissa, 2023 [62]	Saudi Arabia	2023–2023	Online survey	245	≥ 2	- Sleeve gastrectomy - diabetes mellitus, anaemia, - preeclampsia, or hypertension	6	- Preterm birth, - SGA, - LGA, - NICU admission, - jaundice, - birthweight
Feig, 2022 [59]	Canada	2011–2018	Medical records	460	2	- Nephropathy - hypertension	7	- SGA, - birth weight, - gestational age, - preterm birth, - NICU admission within 24 h of birth, - neonatal hypoglycaemia requiring IV dextrose infusion, - head and abdominal circumferences
Huet, 2018 [33]	France	2012–2014	Medical records	1,484	2	- Gestational diabetes mellitus - obesity	15	- Preterm birth (32–37 weeks), - extreme preterm birth (< 32 weeks), - LGA, - macrosomia, - intrauterine growth restriction, - shoulder dystocia, - foetal traumatism, - stillbirth, - early neonatal mortality, - need for ventilation, - admission in neonatology, - Apgar score < 10 at 5 min, - umbilical pH < 7.20, - hypoglycaemia, - hypocalcaemia
Lin, 2024 [38]	China	2015–2022	Medical records	13,645	2	- Gestational diabetes mellitus - hypertensive disorders of pregnancy	5	- Gestational week of delivery, - birthweight, - composite pregnancy outcomes (at least one of: preterm -- birth, placenta previa, or neonatal jaundice), - SGA, - LGA
Liu, 2020 [39]	China	2014–2018	Medical records	2,146	2	- Gestational diabetes mellitus - echocardiographic epicardial adipose tissue	5	- LGA, - neonatal hypoglycaemia, - NICU admission, - preterm delivery, - hyperbilirubinemia
Liu, 2021 [40]	China	2010–2019	Registry	19,424	2	- Coronary heart disease - pulmonary hypertension	4	- Foetal distress, - foetal growth restriction, - foetal malformation, - LBW
Manoharan, 2020 [42]	Australia	2015–2019	Medical records	1,545	2	- Polycystic ovary syndrome (PCOS) - gestational diabetes mellitus	9	- Prematurity, - Apgar score < 7 at 1 min, - Apgar score < 7 at 5 min, - neonatal birth weight, - NICU admission, - neonatal hypoglycaemia, - NICU admission with neonatal hypoglycaemia, - neonatal death, - congenital abnormality
Peyvandi, 2020 [43]	USA	2007–2012	Registry	2,419,651	≥ 2	- BMI > 25 kg/m2, - hypertensive disorder, - diabetes mellitus	3	- CHD, - CHD without chromosomal anomalies, - CHD without single ventricle
Schlichting, 2019 [47]	USA	2008–2013	Registry	22,881,691	2	- Coronary heart disease - pulmonary hypertension	4	- Foetal distress, - foetal growth restriction, - foetal malformation, - foetal death or stillbirth
Sweeney, 2024 [49]	USA	2013–2021	Medical records	40,840	2	- Chronic hypertension - hypertensive disorders of pregnancy	4	- Preterm birth, - SGA, - NICU admission, - perinatal morbidity
Tanner, 2022 [50]	Australia	2009–2017	Registry	17,824	≥ 2	- Preeclampsia - chronic hypertension, - diabetes or obesity	5	- Gestational age at delivery (28–32 weeks, 33 − 26 weeks, >37 weeks), - RDS, - neonatal sepsis, - NICU admission, - Apgar score < 5 at 5 min
Tsur, 2017 [51]	USA	2007–2011	Medical records	2,049,196	≥ 2	- Overweight or obesity - diabetes mellitus, - chronic hypertension, or pregnancy-related hypertensive disorder	1	- Preterm birth
Venkatesh, 2020 [53]	USA	2002–2008	Registry	2,217	2	- Preeclampsia with severe features - chronic hypertension or diabetes mellitus	6	- NICU admission, - gestational age < 28 weeks, 28–32 weeks, 32–34 weeks, - mean gestational age, - mean neonatal length of stay
Wu, 2024 [55]	China	2021–2022	Interviews and medical records	149	2	- PCOS - gestational diabetes mellitus	3	- Apgar score at 1-minute, - neonatal birth weight, - hypoglycaemia
Deutsch, 2022 [57]	USA + South Africa	Not reported	Survey	3,042	2	- Anxiety - depression	2	- Infant neurological maturation, - preterm birth
Männistö, 2016 [41]	USA	2002–2008	Medical records	228,668	≥ 2	- Depression - anxiety or bipolar disorder with depression and/or anxiety	1	- Preterm birth
Miller, 2021 [60]	USA	2017–2021	Interviews	1,274	≥ 3	- Adverse childhood experience score > 3	1	- Preterm birth
Popovic, 2018 [64]	Italy	2005–2016	Survey	5,150	≥ 2	- Eating disorders with depression and/or anxiety	1	- Infant wheezing
Uguz, 2019 [52]	Turkey	Not reported	Interviews and medical records	1,119	2	- Major depression - anxiety disorder	4	- Gestational age at birth, - birthweight, - preterm birth, - LBW
Clements 2016 [61]	USA	2006–2009	Registry	221,860	≥ 2	- Diseases of the circulatory system, musculoskeletal system and connective tissues, nervous system and senses, hypertension, or diabetes, and mental illness	3	- Preterm birth, - SGA, - Apgar score > 7 at 5 min
Flynn 2015 [63]	USA	2010–2012	Registry	419	≥ 3	- Kidney problems, - high blood pressure, or diabetes - mental health status	2	- Birth weight, - Apgar score at 5 min
Jiao 2020 [35]	China	2015–2018	Survey and medical records	4,380	2	- Gestational diabetes mellitus - depression	6	- Gestational age (weeks), - birthweight (g), - body length (cm), - head circumference (cm), - chest circumference (cm), - LGA
Kolstad 2015 [65]	Norway	1999–2018	Registry	107,214	2	- Epilepsy - an eating disorder	6	- Small Ponderal Index (PI), - large PI, - SGA, - LGA, - small head for gestational age, - Apgar score < 7 at 5 min
Wang 2023 [54]	China	2021–2022	Interviews and medical records	5,734	2	- Gestational diabetes mellitus - depression	5	- Gestational age at birth, - preterm birth, - birthweight, - SGA, - LGA
Karasek 2021 [36]	USA	2020–2021	Medical records	240,157	≥ 2	- Covid-19 - hypertension, - diabetes mellitus, or obesity	2	- Very preterm (< 32 weeks), - preterm (< 37 weeks)
Baguiya 2021 [22]	43 countries (LICs + LMICs)	Dec 2017	Medical records	1,219	≥ 2	- Infection of any kind - anaemia, - diabetes, HIV or any disease that required corticotherapy, - chemotherapy, immunotherapy, or transfusion during the pregnancy	4	- Neonatal near miss (birthweight < 1750 g, - gestational age at birth between 28 and 33 weeks, - perinatal death
DeWaard 2021 [58]	South Africa	2020–2020	Registry	100	2	- Covid-19 infection - HIV	2	- Stillbirth, - preterm birth
Guida 2022 [70]	Brazil	2020–2021	Medical records	729	2	- Covid-19 infection - preeclampsia	7	- Preterm birth, - LBW, - Apgar < 7 at 5 min, - neonatal respiratory distress, - NICU admission, - neonatal morbidity, - neonatal death
Nakanishi 2023 [34]	Japan	2011–2014	Registry	86,885	≥ 2	- Allergic diseases, - asthma, - anaemia, - diabetes mellitus, - dyslipidaemia, - epilepsy, - gastric or duodenal ulcer, - heart disease, - hepatitis, - HIV infection, - hypertension, - inflammatory bowel disease, - kidney disease, - malignancy, migraine, - neurological disease, - sexually transmitted diseases, - psychiatric disorders, - rheumatic or collagen diseases, - thyroid disease, - underweight and obesity, - physical or verbal domestic violence, - substance abuse	6	- Extremely low birth weight (< 1000 g), - low birth weight (< 2500 g), - preterm birth (34–37 weeks), - SGA, - very low birth weight (< 1500 g), - preterm birth (before 34 weeks)
Lopez 2019 [67]	USA	2008–2010	Registry	178,737	≥ 2	- Reports scores from the Charlson-Deyo Index for comorbidities [74]	2	- Poor foetal growth, - preterm birth
Priyadharshini 2021 [44]	India	2020–2020	Medical records	381	≥ 2	- Covid-19 - pregnancy induced hypertension, - hypothyroidism, - gestational diabetes mellitus, - anaemia, - cardiovascular disease, - immunocompromised, - kidney disease, or central nervous system disease	1	- Foetal death
Sania 2017 [45]	South Africa	2013–2014	Registry	571	≥ 2	- HIV - depression, - intimate partner violence, - moderate psychological distress, - hazardous alcohol use	2	- SGA, - preterm birth
Sardinha 2024 [46]	Brazil	2020–2021	Medical records	729	2	- Covid-19 infection - overweight or obese	4	- Gestational age at birth, - NICU admission, - neonatal death, - Apgar score at 5 min
Schapkaitz 2021 [68]	South Africa	2017–2020	Medical records	768	2	- HIV - venous thromboembolism	1	- Preterm birth
Sobieray 2024 [48]	Brazil	2020–2021	Medical records	100	2	- Covid-19 infection - preeclampsia	7	- Foetal growth restriction, - foetal death, - prematurity, - birth weight, - LBW, - Apgar score < 7 at 5 min, - NICU admission
Buteau 2023 [26]	Canada	2000–2016	Registry	1,342,198	≥ 2	- Exposure to PM2.5 or NO2 - preexisting diabetes mellitus, - obesity, - preeclampsia, - preexisting hypertension, epilepsy, mood disorders, anaemia, or substance use disorders	1	- CHD (tetralogy of Fallot, coarctation of the aorta, transposition of great vessels, truncus arteriosus, hypoplastic left heart, common ventricle, atrial septal defect, ventricular septal defect, endocardial cushion defect, pulmonary artery defects, or heterotaxy)
Lavigne 2016 [37]	Cananda	2005–2012	Registry	818,400	≥ 2	- Exposure to ambient air pollution - asthma, - hypertension, - heart disease, - diabetes, - gestational diabetes mellitus, or preeclampsia	3	- Preterm birth, - SGA, - LBW
Zhao 2020 [71]	Mongolia	2013–2016	Medical records	69,945	≥ 2	- Exposure to SO2, CO, PM10, O3, NO2, or PM2.5 - gestational diabetes mellitus, - gestational hypertension, - preeclampsia, - polyhydramnios, - oligohydramnios, - hyperthyroidism, - hypothyroidism, or anaemia	2	- Preterm birth, - LBW
Avalos 2024 [20]	USA	2011–2020	Registry	364,924	3	- Cannabis use during pregnancy - anaemia, diabetes, - abnormal BMI, - asthma, nausea, or mental health conditions	6	- LBW, - SGA, - preterm birth < 34 weeks, - preterm birth < 37 weeks, - NICU admission, - neonatal respiratory support
deAndrade 2025 [31]	USA	2000–2004	Registry	150,775	2	- Tobacco use during pregnancy - diabetes, anaemia, or eclampsia	1	- Congenital malformations

### Definitions of multimorbidity

**Fig. 3:**
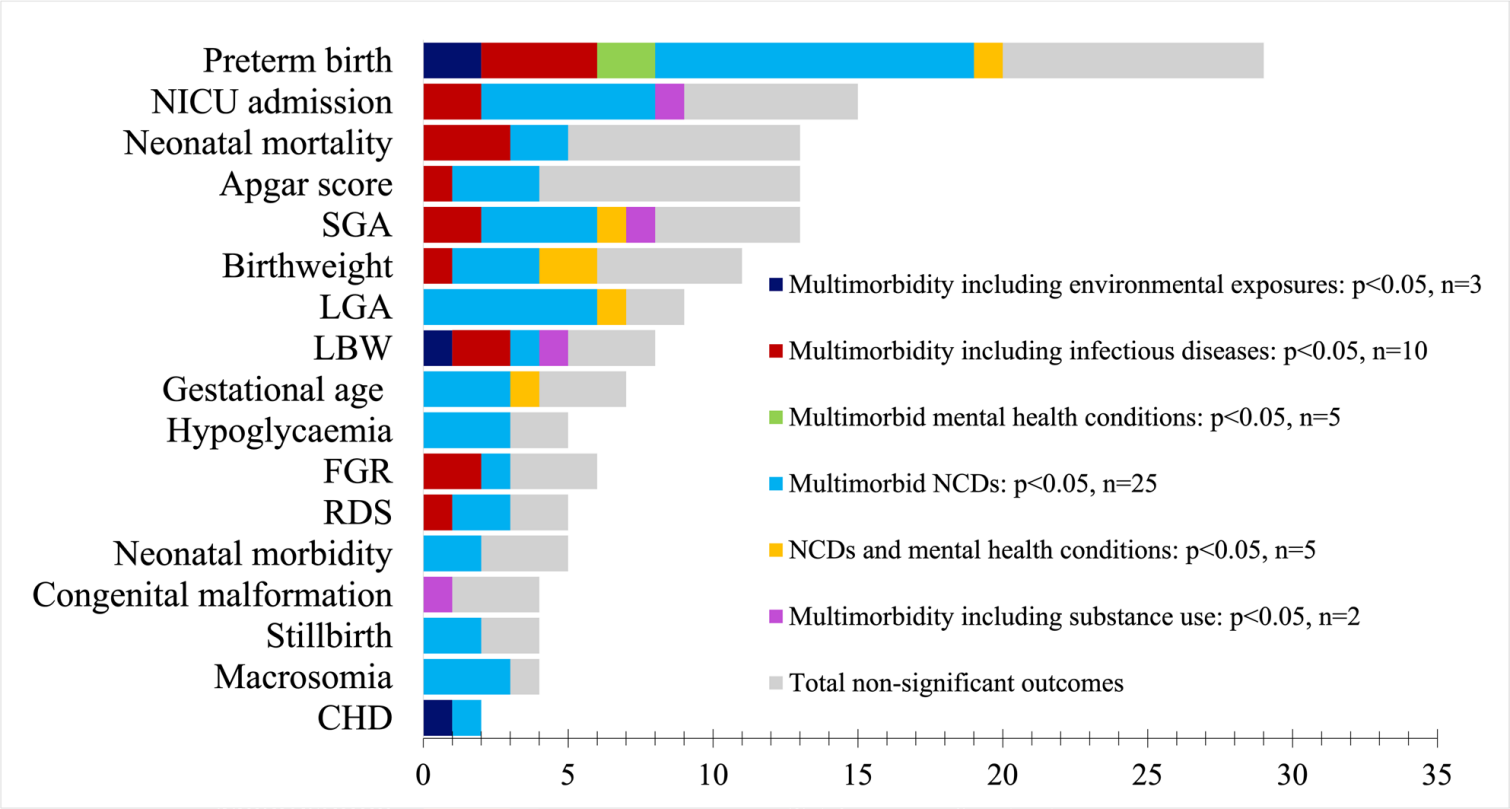
Included papers showing the reported significance of multimorbidity

The included studies covered heterogeneous groups of multimorbidity including varying definitions of multimorbidity during pregnancy shown in Table 3 [see Additional file 4]. Most studies included participants with at least two coexisting conditions during pregnancy (*n* = 50) while four studies set the minimum number of conditions for inclusion to three [19, 20, 60, 63]. The studies also varied in the types of conditions that constituted multimorbidity. Among the publications identified, only two reported a 12-month follow-up. One study investigated multimorbidity during pregnancy, including conditions such as eating disorders, depression, and/or anxiety, and reported infant wheezing [64]. Another study reported on infant neurological maturation within the first year of life [57]. The number of papers in each category of multimorbidity, as well as the papers reporting statistical significance (*p* value < 0.05) of the results, is presented in Fig. 3.

This figure presents the outcomes recorded in each study, categorised by the type of multimorbidity explored. The following outcomes were reported to be significantly associated with multimorbidity in at least one study: neonatal near miss, delayed infant neurological maturation, infant wheezing, hyperbilirubinemia, and sepsis. The following outcomes showed no significant association with multimorbidity in any studies: arterial cord pH < 7.1, head circumference, chest circumference, body length, hypoglycaemia, hypoxic ischaemic encephalopathy, foetal distress, large ponderal index, small ponderal index, clavicle fracture, cardiopulmonary resuscitation, enterocolitis, intraventricular haemorrhage, jaundice, abdominal circumference, shoulder dystocia, foetal traumatism, hypocalcaemia, and need for ventilation. Studies reporting prevalence without assessing significance were excluded from this figure [18, 53, 58, 69]. Abbreviations: Neonatal intensive care unit (NICU), small-for-gestational age (SGA), large-for-gestational age (LGA), low birth weight (LBW), foetal growth restriction (FGR), respiratory distress syndrome (RDS), congenital heart disease (CHD).

### Multimorbid NCDs

Most studies (*n* = 28) reported on pregnant women with multimorbidity of two or more NCDs [18, 19, 21, 23–25, 27–30, 32, 33, 38–40, 42, 43, 47, 49–51, 53, 55, 56, 59, 62, 66, 69]. These conditions included chronic conditions, such as diabetes and obesity, and conditions that arise during pregnancy, such as preeclampsia and gestational diabetes.

Measures of birthweight (i.e., SGA, LGA FGR, macrosomia and birthweight averages) were reported in fifteen studies [19, 25, 28, 29, 32, 33, 38–40, 47, 49, 55, 56, 59, 62]. Fifteen studies also reported preterm birth as an outcome of interest [18, 19, 21, 23, 24, 28, 29, 33, 38, 39, 49, 51, 59, 62, 66]. The next most common outcome reported was NICU admission (*n* = 14) [18, 19, 24, 25, 27, 32, 39, 42, 49, 50, 53, 56, 59, 62]. Neonatal death was reported by 10 studies [18, 19, 24, 25, 30, 32, 42, 47, 56, 69] and Apgar score was reported by 8 studies [19, 27, 33, 42, 50, 55, 56, 69]. Neonatal hypoglycaemia was reported in six studies [19, 33, 39, 42, 55, 59]. Stillbirth was reported in five studies [18, 30, 32, 33, 47]. Twenty-one studies had significant findings (*p* < 0.05) for the association between multimorbidity during pregnancy and at least one adverse outcome [19, 21, 24, 25, 28–30, 32, 33, 38–40, 42, 43, 49–51, 55, 56, 59, 62]. Three studies reported only on prevalence within the study group and did not calculate significance for the reported outcomes [18, 53, 69]. Four studies found no significant relationship between multimorbidity and the adverse neonatal outcomes investigated [23, 27, 47, 66].

### Multimorbidity and mental health conditions

Five studies reported outcomes for pregnant women with two or more coexisting mental health conditions or psychological morbidities [41, 52, 57, 60, 64]. These conditions of interest included anxiety, depression, post-traumatic stress disorders, and eating disorders. The most common outcome reported in this group was preterm birth, which was reported by four studies [41, 52, 57, 60]. Neurological maturation, infant wheezing, gestational age at birth, and abnormal birthweight were each reported once [52, 57, 64]. All five studies found significant results between comorbid mental health disorders during pregnancy and adverse neonatal outcomes.

### NCDs and mental health disorders

Five studies reported outcomes from pregnant women with both NCDs and psychological morbidities or mental health disorders [35, 54, 61, 63, 65]. The most common outcomes reported for these comorbidities were abnormal birth weight, Agar scores, and preterm birth. Birthweight measures such as SGA, LGA, and birthweight averages were measured by all five studies. Three studies reported Apgar scores [61, 63, 65] and two studies reported preterm birth [54, 61]. Three of these studies found a significant relationship between multimorbidity during pregnancy and the outcomes that were assessed [35, 61, 63]. However, two studies found no significance [54, 65].

### Infectious diseases with comorbid conditions

Eleven studies included pregnant women with an infectious disease and a comorbid chronic condition, including both psychological morbidities and NCDs [22, 34, 36, 44–46, 48, 58, 67, 68, 70]. The most common outcome reported was preterm birth, which was included in nine studies [22, 34, 36, 45, 48, 58, 67, 68, 70]. This was followed by perinatal death and measures of birthweight (including low birth weight, SGA and poor foetal growth), reported by five papers [34, 45, 48, 67, 70]. Both Apgar scores and NICU admission were reported by three papers [46, 48, 70]. Of the eleven studies in this category, nine studies reported significant outcomes linking multimorbidity during pregnancy to adverse outcomes [22, 34, 36, 45, 46, 48, 67, 68, 70]. One study reported that a significant relationship [44] was not found and another reported prevalence without an indication of significance [58].

### Environmental exposures with multimorbidity

Three studies included environmental exposures [26, 37, 71] in the definition of multimorbidity, including exposure to substances that have been proven harmful to human health, such as ambient air pollution, sulphur dioxide, carbon monoxide, nitrogen dioxide, and other particulate matter. All three studies reported significant results for outcomes such as preterm birth, abnormal birth weight, and the development of congenital heart diseases. Preterm birth and measures of birthweight were both reported in two of the three studies [37, 71]. The third study reported the presence of congenital heart disease including conditions such as septal defects, coarctation of the aorta, and heterotaxy [26].

### Substance use and multimorbidity

Two papers included substance use during pregnancy combined with NCDs or mental health conditions in the definition of multimorbidity [20, 31]. Both studies reported significant associations between multimorbidity, and the adverse perinatal outcomes investigated. One study assessed the use of cannabis products with comorbidities such as anaemia, diabetes, or mental health conditions and reported outcomes including abnormal birthweight, preterm birth, NICU admission, and need for respiratory support [20]. The second paper studied tobacco use and diabetes, anaemia, or preeclampsia during pregnancy and associated congenital malformations [31].

## Discussion

This systematic review synthesised evidence from 54 studies from 20 countries and across eight regions to examine the relationship between multimorbidity during pregnancy and perinatal and neonatal outcomes. A key finding is the substantial heterogeneity in the definition of multimorbidity in pregnancy across studies, with most (*n* = 46), including two or more coexisting conditions, but some using three or more as criteria. The range of conditions considered constitute broad categories including NCDs (*n* = 28), infectious diseases (*n* = 11), coexisting mental health conditions and NCDs (*n* = 5) mental health conditions (*n* = 5), environmental exposures (*n* = 3), and substance use (*n* = 2). These conditions yielded varied adverse outcomes, such as low birth weight, SGA, intrauterine growth restriction, and low Apgar scores, which may reveal insight into the impact each cluster of multimorbid conditions has on foetal development. However, methodological inconsistencies limited comparability across studies; not all studies reported significance testing for observed outcomes, few (*n* = 4) only presented prevalence data, and a minority found no statistically significant associations with multimorbidity during pregnancy.

The studies included in this systematic review attest to differing research priorities by country, region, and income level. Studies investigating infectious diseases with at least one comorbidity during pregnancy were primarily conducted in middle and lower-income countries in Sub-Saharan Africa, Latin America and the Caribbean, and Central and Southern Asia. The global prevalence of multimorbidity during pregnancy is still unclear, partly due to limited diagnostic capabilities in lower socioeconomic settings. The skewed availability of information from HICs resulted in the current evidence landscape is not globally representative. Most of the included studies (66%) were conducted in high-income settings, predominantly in Northern America (37%) and Europe (17%), while 35% were conducted in middle-income countries, and only one study in LIC. This geographical and socioeconomic concentration highlights imbalance in evidence generation, reflecting broader inequalities in global maternal and child health research. The overrepresentation of HIC settings likely limit the applicability of findings to populations in LMICs most affected by maternal multimorbidity, where health system constraints, social determinants, and patterns of disease coexistence may differ substantially [75].

The included studies also varied in the types of conditions that constituted multimorbidity; for example, some included pre-eclampsia and liver dysfunction [18] for multimorbid NCDs, whereas others included pre-eclampsia and acute kidney injury [30]. Definitions of multimorbidity during pregnancy varied by the number of conditions present and the types of conditions included— e.g., substance use, environmental exposures — which limited the ability to draw conclusions across studies. In line with previous research [9, 76], this review reveals the need for standardised definitions to facilitate future research analysed on a global scale.

Variation in defining multimorbidity extend to differences in how outcomes are defined, measured, and reported across studies. Our review aligns with prior systematic reviews recommending explicit cut-offs (e.g., ≥ 2 conditions), validated weighted measures where possible, and rationales for include or excluded conditions to improve comparability [9, 77]. It is possible that differences in diagnostic criteria, thresholds for classifying conditions, and timing of assessments further contribute to discrepancies, often reflecting methodological heterogeneity rather than true biological differences [78]. Furthermore, women with multimorbidity frequently experience polypharmacy, which can increase the risk of adverse drug interactions, toxicity, and negative effects on the foetus [79]. The results of this systematic review could be used to inform the adoption of an updated international core set of standard conditions to be recorded at birth. The survey of outcomes reported in our systematic review can guide the development of standard measurement protocols, ensuring consistency in included conditions, the cutoff values for inclusion (such as the weight at birth and gestational age), and the time at which measurements are taken.

Overall, preterm birth, NICU admission, measure of birthweight (including SGA, LGA and birthweight averages), Apgar scores, and neonatal death were reported more than any other outcomes. Most studies reported a significant relationship between multimorbidity during pregnancy and preterm birth, NICU admission, and measures of abnormal birthweight. While some studies found significant results linking multimorbidity during pregnancy to low Apgar scores and neonatal death, most did not find statistically significant results. Surprisingly, we identified only two papers reporting outcomes within the first year after birth. The limited reporting infant outcomes beyond the perinatal period and within the first year after birth; this limited reporting highlights an important evidence gap, as studies prioritise immediate perinatal risks, potentially overlooking the sustained impacts of multimorbidity.

### Policy and practice

There are no current public health policies that specifically address multimorbidity during pregnancy. The World Health Organization (WHO) has published no recommendations for treatment of pregnant women with multiple chronic conditions, and while the National Institute for Health and Care Excellence (NICE) has current guidelines on single conditions during pregnancy and multimorbidity in the general population [80], there are no recommendations for multimorbidity during pregnancy. Elements from the WHO guidelines for treatment of single conditions during pregnancy and the NICE guidelines on multimorbidity in the general population should be combined to develop guidelines for managing the most common multimorbidity clusters.

Policies promoting prenatal care and routine screening of diseases in that are commonly clustered in multimorbidity may reveal conditions that would have otherwise been left undiagnosed. Identifying multimorbidity women of reproductive age is crucial for deciding future treatments throughout pregnancy and postpartum. Guidelines should be drafted for multimorbidity during pregnancy using recommendations and guidance from healthcare providers due to the complexity of this topic. Further, resources should be allocated to primary care physicians to give them a leadership role in multimorbidity care. Rather than referring patients to multiple specialists, these providers can help develop a patient-centred treatment plan, working closely with specialists, including obstetricians or midwives, and overseeing the care provided to coordinate a cohesive plan. Transitioning from a system that uses multiple providers to target individual conditions into a team-based approach may reduce problems associated with multimorbidity, such as polypharmacy and adverse interactions between treatments [78]. Interventions should be tailored to the individual, considering the socioeconomic contexts, unique conditions, and goals for treatment to be effective. Addressing multimorbidity during pregnancy is complex, but access to diagnostic technology and integrative care may better the health of the mother and reduce the risk of adverse outcomes in offspring.

### Strengths and limitations

To the best of our knowledge, this is the first systematic review to assess the impact of multimorbidity on neonatal and perinatal outcomes through the first year of life. This fills a significant research gap as multimorbidity during pregnancy is an under-researched topic, yet this subject greatly affects maternal morbidity and under five mortality rates. Likewise, the neonatal and perinatal outcomes of pregnancies from women with multimorbidity were not specified or limited in the search strategy, allowing for a wide scope of search. This allowed a survey of the reported outcomes and definitions of key terms, such as multimorbidity, perinatal outcomes, and neonatal outcomes, to be conducted. Limiting the inclusion to a specific definition or outcome would have narrowed the scope of the systematic review and reduced the external validity of the review.

This systematic review included many studies for analysis, while limiting to publication within the last ten years, and the papers in this review reflect the most current information available on the topic. Overall, the studies included in this systematic review were of good quality, with no studies scoring under a six on the NOS. These scores reveal that none of the included studies were at a high risk of bias in their results and reduce the chance that the conclusions drawn in this review are inaccurate. The quality of a review is only as high as the data sources that it is based upon, so this systematic review is strengthened by the quality of the included articles.

This systematic review also has limitations that must be considered. The definitions of multimorbidity varied within the included studies, and a diverse mix of adverse outcomes were reported, which produced heterogeneity between the included studies. The variability across studies prevented us from conducting a meta-analysis or subgroup analysis or assess the overall pooled estimate of the effect of multimorbidity on perinatal and neonatal outcomes. Exclusion of studies fewer than 100 participants (*n* = 32) minimised bias from low statistical power but may have omitted relevant data from low-resource settings where smaller studies predominate. No grey literature search was conducted, potentially missing unpublished studies or non-indexed sources that could address publication bias. Most included studies originated from HIC, limiting global representativeness given scarce data from LMICs, where diagnostic and funding constraints hinder large-scale research. Further studies are required from LMICs to elucidate the global burden or maternal multimorbidity and its perinatal impacts, emphasising both statistical and clinical relevance. There is also a need for further research to comprehensively address both statistical significance and clinical relevance, thereby enhancing the practical applicability of the results.

## Conclusion

This review synthesised literature describing the adverse perinatal and neonatal outcomes of multimorbidity during pregnancy. The 54 studies included in this review were conducted in different global regions and income levels, showing variance in the multimorbid conditions found in the study populations, the outcomes of interest, and how these outcomes were reported. Substantial heterogeneity in the definition and measurement of multimorbidity complicates comparisons, yet the evidence consistently indicates that multimorbidity in pregnancy is associated with adverse outcomes—particularly preterm birth, NICU admission, and abnormal weight. Associations with Apgar scores and neonatal mortality were less conclusive, reflecting inconsistent reporting. Importantly, no study identified a protective effect of multimorbidity. The findings identify a substantial burden of adverse perinatal and neonatal outcomes associated with multimorbidity during pregnancy and highlight the need for standardised definitions for multimorbidity and perinatal outcomes and expanded research in understudied regions to better understand and address the burden of maternal multimorbidity. Furthermore, leveraging routine health information systems to improve multimorbidity surveillance during pregnancy; and examining long-term maternal and child outcomes beyond the first year of life.

## Supplementary Information

Additional file 1.

Additional file 2.

Additional file 3.

Additional file 4.

## Data Availability

The list of included studies is provided in Table 2. No new datasets were created as all information is derived from previously published studies.

## Abbreviations

CHD: congenital heart disease
FGR: foetal growth restriction
HIC: high-income country
LIC: low-income country
LGA: large-for-gestational age
LMIC: lower middle-income countries
NCDs: non-communicable diseases
NICE: National Institute for Health and Care Excellence
NICU: neonatal intensive care unit
NOS: Newcastle-Ottawa Scale
RDS: respiratory distress syndrome
SGA: small-for-gestational age
UMICs: upper middle-income countries
UN: United Nations
WHO: World Health Organization
