# Supplementary material for: Perinatal and neonatal outcomes among women with multimorbidity during pregnancy globally: a systematic review
Source: BMC Pediatr. 2026 Mar 18;26:282. doi: 10.1186/s12887-026-06706-9 (PMC13063708; doi:10.1186/s12887-026-06706-9)
Supplement: Supplementary file 2 — Additional file 2. [file 12887_2026_6706_MOESM2_ESM.docx]

**Supplemental Table 2. Search Strategy**

| Database |  | Search terms | Results |
| --- | --- | --- | --- |
| MEDLINE |  |  |  |
|  | 1 | exp pregnancy/ | 1053151 |
|  | 2 | exp pregnant woman/ | 16231 |
|  | 3 | exp pregnancy outcome/ | 91159 |
|  | 4 | (pregnan* or perinatal or maternal or gestational).ti,ab,kw. | 930613 |
|  | 5 | 1 or 2 or 3 or 4 | 1375578 |
|  | 6 | exp multimorbidity/ | 3753 |
|  | 7 | exp multiple chronic conditions/ | 794 |
|  | 8 | exp comorbidity/ | 133337 |
|  | 9 | (comorbid* or "multiple long-term conditions" or multimorbid* or co-morbid* or multi-morbid*).ti,ab,kw. | 310648 |
|  | 10 | 6 or 7 or 8 or 9 | 381357 |
|  | 11 | exp risk factors/ | 1025615 |
|  | 12 | (factor* or determinant* or pattern* or association* or predictor*).ti,ab,kw. | 7610729 |
|  | 13 | 11 or 12 | 7975257 |
|  | 14 | 5 and 10 and 13 | 6446 |
|  | 15 | limit 14 to yr="2015-current" | **4181** |
| Embase |  |  |  |
|  | 1 | 'pregnancy'/exp | 932961 |
|  | 2 | 'pregnant woman'/exp | 128708 |
|  | 3 | 'pregnancy outcome'/exp | 93145 |
|  | 4 | pregnan*:ti,ab,kw OR maternal:ti,ab,kw OR gestational:ti,ab,kw | 1228939 |
|  | 5 | 1 or 2 or 3 or 4 | 1560230 |
|  | 6 | 'multiple chronic conditions'/exp | 11335 |
|  | 7 | 'comorbidity'/exp | 448844 |
|  | 8 | 'multiple chronic*:ti,ab,kw OR comorbid*:ti,ab,kw OR multimorbid*:ti,ab,kw OR 'co-morbid*':ti,ab,kw OR 'multi-morbid*':ti,ab,kw OR 'multiple long-term*':ti,ab,kw | 549576 |
|  | 9 | 6 or 7 or 8 | 698976 |
|  | 10 | risk factor/exp | 1515824 |
|  | 11 | factor*:ti,ab,kw OR determinant*:ti,ab,kw OR pattern*:ti,ab,kw OR association*:ti,ab,kw OR predictor*:ti,ab,kw | 10079004 |
|  | 12 | 10 or 11 | 10513565 |
|  | 13 | 5 and 9 and 12 | 11131 |
|  | 14 | 13 and [2015-2025]/py and [embase]/lim | 7802 |
|  | 15 | 13 and [2015-2025]/py and [embase]/lim and [conference abstract]/lim | 2690 |
|  | 16 | 14 not 15 | **5112** |
| Web of Science |  |  |  |
|  | 1 | TS=(pregnan* or "pregnancy outcome*" or perinatal or maternal or gestational)) | 1065421 |
|  | 2 | TS=("multiple chronic*" or comorbid* or multimorbid* or co-morbid* or multi-morbid* or "multiple long-term") | 358527 |
|  | 3 | TS=(determinant* or pattern* or association* or predictor* or "risk factor*") | 7684149 |
|  | 4 | #1 AND #2 AND #3 | 4816 |
|  | 5 | #1 AND #2 AND #3 and 2025 or 2024 or 2023 or 2022 or 2021 or 2020 or 2019 or 2018 or 2017 or 2016 or 2015 (Publication Years) | **3714** |
