# Supplementary material for: Perinatal and neonatal outcomes among women with multimorbidity during pregnancy globally: a systematic review
Source: BMC Pediatr. 2026 Mar 18;26:282. doi: 10.1186/s12887-026-06706-9 (PMC13063708; doi:10.1186/s12887-026-06706-9)
Supplement: Supplementary file 3 — Additional file 3. [file 12887_2026_6706_MOESM3_ESM.docx]

## **Supplemental Table 3: Newcastle-Ottawa Scale for Cohort and Cross-sectional Studies**

| Cohort  studies | Selection | | | | Comparability | Outcomes | |  |  |
| --- | --- | --- | --- | --- | --- | --- | --- | --- | --- |
| Study | Representativeness of the exposed  cohort | Selection of the non-  exposed  cohort | Ascertainment of  exposure | Demonstration that outcome of interest was not present at start of study | Comparability of the study controls  (multimorbidity during pregnancy) for age, sex or other sociodemographic factors | Assessment of  neonatal/ perinatal outcomes | Was follow-up long enough for outcomes to occur | Adequacy of follow-up of  cohorts | Total |
| Allen 2016 (18) | * | * | * | * | ** | * | * | * | 9* |
| Aubry 2019 (19) | * | * | * | * | ** | * | * | * | 9* |
| Avalos 2024 (20) | * | * | * | * | ** | * | * | * | 9* |
| Azcoaga-Lorenzo  2023 (21) | * | * | * | * | ** | * | * | * | 9* |
| Baguiya 2021 (22) | * | * | * | * | ** | * | * | * | 9* |
| Bapayeva 2022 (23) | * | * | * | * | ** | * | * | * | 9* |
| Belsti 2024 (24) | * | * | * | * | ** | * | * | * | 9* |
| Brakewood 2024 (25) | * | * | * | * | ** | * | * | * | 9* |
| Buteau 2023 (26) | * | * | * | * | ** | * | * | * | 9* |
| Chaalan 2024 (27) | * | * | * | * | ** | * | * | * | 9* |
| Chen 2022 (28) | * | * | * | * | ** | * | * | * | 9* |
| Chen 2023 (29) | * | * | * | * | ** | * | * | * | 9* |
| Clements 2016 (61) |  | * | * | * | ** | * | * |  | 7* |
| Conti-Ramsden  2019 (30) | * | * | * | * | ** | * | * | * | 9* |
| Cozzi 2024 (56) |  | * | * | * | ** | * | * | * | 8* |
| De Andrade 2025 (31) | * | * | * | * | ** | * | * | * | 9* |
| Deutsch 2022 (57) | * | * | * | * | * | * | * | * | 8* |
| DeWaard 2021 (58) |  | * | * | * | ** | * | * | * | 8* |
| Dudley 2017 (32) | * | * | * | * | ** | * | * | * | 9* |
| Eissa 2023 (62) | * | * |  | * | ** |  | * | * | 7* |
| Feig 2022 (59) |  | * | * | * | ** | * | * | * | 8* |
| Flynn 2015 (63) |  | * | * | * | ** | * | * |  | 7* |
| Huet 2018 (33) | * | * | * | * | ** | * | * | * | 9* |
| Nakanishi 2023 (34) | * | * | * | * | ** | * | * | * | 9* |
| Jiao 2020 (35) | * | * | * | * | ** | * | * | * | 9* |
| Karasek 2021 (36) | * | * | * | * | ** | * | * | * | 9* |
| Kolstad 2015 (65) | * | * |  | * |  | * | * | * | 6* |
| Lavigne 2016 (37) | * | * | * | * | ** | * | * | * | 9* |
| Lin 2024 (38) | * | * | * | * | ** | * | * | * | 9* |
| Liu 2020 (39) | * | * | * | * | ** | * | * | * | 9* |
| Liu 2021 (40) | * | * | * | * | ** | * | * | * | 9* |
| Männistö 2016 (41) | * | * | * | * | ** | * | * | * | 9* |
| Manoharan 2020 (42) | * | * | * | * | ** | * | * | * | 9* |
| Miller 2021 (60) | * | * |  | * | ** | * | * | * | 8* |
| Peyvandi 2020 (43) | * | * | * | * | ** | * | * | * | 9* |
| Popovic 2018 (64) | * | * |  | * | ** |  | * | * | 7* |
| Priyadharshini 2021 (44) | * | * | * | * | ** | * | * | * | 9* |
| Sania 2017 (45) | * | * | * | * | ** | * | * | * | 9* |
| Sardinha 2024 (46) | * | * | * | * | ** | * | * | * | 9* |
| Schlichting 2019 (47) | * | * | * | * | ** | * | * | * | 9* |
| Sobieray 2024 (48) | * | * | * | * | ** | * | * | * | 9* |
| Sweeney 2024 (49) | * | * | * | * | ** | * | * | * | 9* |
| Tanner 2022 (50) | * | * | * | * | ** | * | * | * | 9* |
| Tsur 2017 (51) | * | * | * | * | ** | * | * | * | 9* |
| Uguz 2019 (52) | * | * | * | * | ** | * | * | * | 9* |
| Venkatesh 2020 (53) | * | * | * | * | ** | * | * | * | 9* |
| Wang 2023 (54) | * | * | * | * | ** | * | * | * | 9* |
| Wu 2024 (55) | * | * | * | * | ** | * | * | * | 9* |

| Cross-  sectional  studies | Selection | | |  | Comparability | Outcomes |  | |  |
| --- | --- | --- | --- | --- | --- | --- | --- | --- | --- |
| Study | Is the case  definition  adequate? | Representativeness of the cases | Selection of controls | Definition of controls | Study controls for newborn/child  outcomes and any additional factors | Ascertainment of exposures | Same method of ascertainment for case control trials | Non-  response rate | **Total** |
| Bartáková 2017 (69) | * |  | * | * | ** | * | * |  | 7* |
| Bröms 2016 (66) | * | * | * | * | ** | * | * | * | 9* |
| Guida 2022 (70) |  | * |  | * | ** | * | * | * | 7* |
| Lopez 2019 (67) | * | * | * | * | ** | * | * | * | 9* |
| Schapkaitz 2021 (68) | * | * |  | * | ** | * | * | * | 8* |
| Zhao 2020 (71) |  | * |  | * | ** | * | * |  | 6* |
