# Supplementary material for: Perinatal and neonatal outcomes among women with multimorbidity during pregnancy globally: a systematic review
Source: BMC Pediatr. 2026 Mar 18;26:282. doi: 10.1186/s12887-026-06706-9 (PMC13063708; doi:10.1186/s12887-026-06706-9)
Supplement: Supplementary file 4 — Additional file 4. [file 12887_2026_6706_MOESM4_ESM.docx]

## **Table 3: Newcastle-Ottawa Scale for cross-sectional studies**

| Cross-  sectional  studies | Selection | | |  | Comparability | Outcomes |  | |  |
| --- | --- | --- | --- | --- | --- | --- | --- | --- | --- |
| Study | Is the case  definition adequate? | Representativeness of the cases | Selection of controls | Definition of controls | Study controls for newborn/child  outcomes and any  additional factors | Ascertainment of exposures | Same method of ascertainment for case control trials | Non-  response rate | Total |
| Bartáková 2017 | * |  | * | * | ** | * | * |  | 7* |
| Bröms 2016 | * | * | * | * | ** | * | * | * | 9* |
| Guida 2022 |  | * |  | * | ** | * | * | * | 7* |
| Lopez 2019 | * | * | * | * | ** | * | * | * | 9* |
| Schapkaitz 2021 | * | * |  | * | ** | * | * | * | 8* |
| Zhao 2020 |  | * |  | * | ** | * | * |  | 6* |
